# Changes in Health and Well-Being in COVID-19 Clinically Vulnerable Older English People During the Pandemic

**DOI:** 10.1093/geroni/igab046.359

**Published:** 2021-12-17

**Authors:** Giorgio Di Gessa, Debora Price

**Affiliations:** 1 University College London, London, England, United Kingdom; 2 University of Manchester, Manchester, England, United Kingdom

## Abstract

People with specific health profiles and diseases (such as diabetes, lung and heart conditions) have been classified as ‘clinically vulnerable’ (CV) to Covid-19, i.e. at higher risk of severe illness and mortality from Covid-19, and were targeted for shielding. However, there is as yet little evidence on how the pandemic and shielding impacted the health and social well-being of CV older people. Using data from Wave 9 (2018/19) and the first Covid-19 sub-study (June/July 2020) of the English Longitudinal Study of Ageing, we investigated changes in health and well-being during the pandemic by clinical vulnerability. We also explored the interactions between CV and age-group (50s, 60s, 70s, 80+), and between CV and shielding. Results suggest that CV older people (~39% of the sample) were more likely to report worse health and social well-being outcomes during the pandemic compared to non-CV participants, even considering pre-pandemic levels of health and well-being. However, changes in health were not uniform across age groups, with those in their 50s and 60s more likely to report greater deterioration in mental health than those in their 70s and over 80. Moreover, older adults who were shielding and were CV reported the most substantial rises in anxiety, depression, receipt of formal care as well as decreases in well-being and physical activity. While policies focussing on shielding CV older people reduce rates of hospitalisation and death from Covid-19, policymakers should address the wider needs of this group if their long-term health and social well-being are not to be compromised.

